# Non-linear, non-monotonic effect of nano-scale roughness on particle deposition in absence of an energy barrier: Experiments and modeling

**DOI:** 10.1038/srep17747

**Published:** 2015-12-11

**Authors:** Chao Jin, Tomasz Glawdel, Carolyn L. Ren, Monica B. Emelko

**Affiliations:** 1 Department of Civil and Environmental Engineering, University of Waterloo, 200 University Ave W., Waterloo, ON, N2L 3G1, Canada; 2 Xagenic Inc., 55 York Street, Suite 1000, Toronto, Ontario, M5J 1R7, Canada; 3 Department of Mechanical and Mechatronics Engineering, University of Waterloo, 200 University Ave W., Waterloo, ON, N2L 3G1, Canada

## Abstract

Deposition of colloidal- and nano-scale particles on surfaces is critical to numerous natural and engineered environmental, health, and industrial applications ranging from drinking water treatment to semi-conductor manufacturing. Nano-scale surface roughness-induced hydrodynamic impacts on particle deposition were evaluated in the absence of an energy barrier to deposition in a parallel plate system. A non-linear, non-monotonic relationship between deposition surface roughness and particle deposition flux was observed and a critical roughness size associated with minimum deposition flux or “sag effect” was identified. This effect was more significant for nanoparticles (<1 μm) than for colloids and was numerically simulated using a Convective-Diffusion model and experimentally validated. Inclusion of flow field and hydrodynamic retardation effects explained particle deposition profiles better than when only the Derjaguin-Landau-Verwey-Overbeek (DLVO) force was considered. This work provides 1) a first comprehensive framework for describing the hydrodynamic impacts of nano-scale surface roughness on particle deposition by unifying hydrodynamic forces (using the most current approaches for describing flow field profiles and hydrodynamic retardation effects) with appropriately modified expressions for DLVO interaction energies, and gravity forces in one model and 2) a foundation for further describing the impacts of more complicated scales of deposition surface roughness on particle deposition.

The deposition of colloidal- and nano-scale particles on surfaces is critical to numerous natural and engineered environmental, health, and industrial applications. These include provision of safe drinking water by pathogen filtration in the subsurface^1^,^2^ or in engineered filters^3^,^4^, control of chronic contamination of processed food supplies^5^, improved medical screening and treatment^6^,^7^,^8^, pipeline performance assessment^9^, and improved semiconductor manufacturing^10^ to name a few. Despite the wide range of applications reliant upon particle deposition on surfaces, this process remains inadequately described both conceptually and mathematically when deposition surface roughness is present, thereby precluding the development and application of predictive models for particle deposition on surfaces.

Description of the effect of deposition surface roughness on particle deposition is complicated because there are several relative length scales between surface roughness asperities, particle dimensions, and other system characteristics that influence the local flow field and thus particle deposition mechanisms. The lack of understanding of this relationship is evidenced by inconsistent and contradictory experimental observations that have been reported^11^,^12^,^13^,^14^. Deposition surface roughness, with size as small as a few or hundred nanometers, can significantly enhance particle deposition in some cases^15^,^16^,^17^,^18^. For example, Chen *et al.* (2010) coated stainless steel and aluminum alloys with zeolite to increase deposition surface roughness and increased colloid deposition by up to 50%^19^. Darbha *et al.* (2010) reported a positive correlation between calcite surface roughness and the number of attached colloidal particles^20^; they also found that surface roughness-enhanced particle deposition was more significant for smaller particles (0.3 μm), as compared to larger (2 μm) ones^21^. Zan *et al.* (2008) investigated opportunities for enhancing orthopaedic stainless steel affinity to host tissue and also found that more colloidal particles attached on rough deposition surfaces than on smooth ones^22^. Several studies have reported that deposition surfaces can significantly alter the shape and magnitude of particle-surface interaction energy profiles and have attributed enhanced particle deposition to less repulsive force due to the presence of deposition surface roughness^12^,^18^,^23^,^24^,^25^.

In contrast, decreased particle deposition also has been associated with deposition surface roughness. For example, Tang *et al.* (2009) reported that rougher silicon surfaces did not promote bacterial adhesion and colonization when deposition surface roughness size was below a certain threshold (i.e. 200 nm)^26^. Similarly, Chen *et al.* (2010) found that deposition surface roughness resulted in increased particle deposition in most cases; however, two exceptions were noted in which a rough surface had less particle deposition than a smooth surface comprised of the same material and exposed to the same operational conditions^19^. Similar results regarding media surface roughness impacts on particle deposition behavior have also been reported in packed bed filters^16^,^27^,^28^. Although a recent experimental investigation suggested that media surface roughness affects particle deposition in a nonlinear, non-monotonic manner in those systems, that phenomenon has not been mechanistically or quantitatively described^27^. Given the range of seemingly contradictory experimental outcomes regarding deposition surface roughness effects on particle deposition that have been reported, it is not surprising that comprehensive conceptual and quantitative models describing deposition surface roughness impacts on particle deposition have not be developed.

Particle deposition on surfaces generally consists of two steps: particle transport to and attachment on surfaces^29^,^30^. The deposition of colloids under the influence of external forces including Van der Waals (VDW), electrostatic double layer (EDL), gravity, and hydrodynamic retardation can be quantitatively described using both Eulerian and Lagrangian methods. Existing models are able to predict particle deposition on surfaces when the following assumptions are satisfied 1) the colloidal particles and deposition surface are smooth and chemically homogenous, 2) the interaction energy barrier between the approaching particle and the deposition surface is not large, 3) colloid attachment is predominately governed by chemical interactions between particles and deposition surfaces that are independent of the flow field, and 4) there are no particle-to-particle interactions in the suspension or blocking effects on the deposition surface (i.e., “clean bed” period)^31^,^32^,^33^,^34^,^35^. These assumptions are not valid for most applications, thereby rendering most existing models inadequate for describing particle deposition in real systems, with predictions that are frequently off by several orders of magnitude^36^,^37^,^38^,^39^. One reason for this is that most surfaces commonly present in natural and engineered systems are not perfectly smooth, with at least some surface roughness, at the nano-scale or larger. It has been suggested that differences in particle deposition in the presence of deposition surface roughness may be attributable to changes in DLVO interaction energy^12^,^25^, chemical heterogeneity on charged surfaces^40^, hydrophobicity between particles and surfaces^26^, straining^39^, or rolling^8^,^41^. Quantitative evaluations and numerical representations of these hypotheses are lacking, however.

The present investigation focused on experimentally demonstrating and modeling the effect of nano-scale surface roughness on particle deposition in the absence of an energy barrier—a condition common in many engineered filtration applications. The key components of this work included 1) fabricating quartz surfaces with uniform, homogenous nano-scale roughness; 2) conducting rigorous quality assurance experiments to exclude confounding factors and enable incontrovertible demonstration of deposition surface roughness impacts on particle deposition; 3) developing an approach for accurately describing deposition surface roughness features, and most notably, 4) developing the first comprehensive framework for describing the hydrodynamic impacts of nano-scale surface roughness on particle deposition by unifying hydrodynamic forces (using the most current approaches for describing flow field profiles and hydrodynamic retardation effects) with appropriately modified expressions for DLVO interaction energies, and the gravity force, in one model.

## Model development

**Figure 1:**
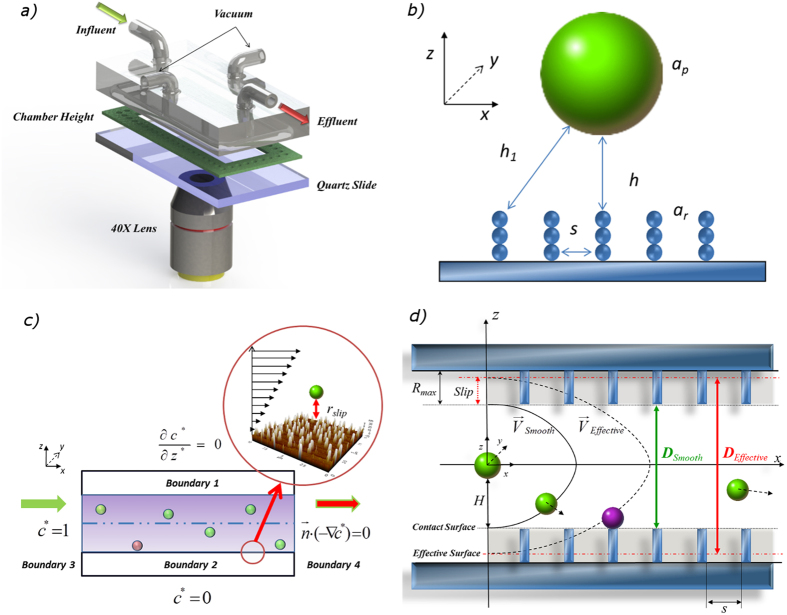
Schematic of (a) experimental parallel plate chamber set-up; (b) geometric conceptualization of rough surfaces; (c) parallel plate chamber boundary conditions; (d) flow field and diffusion coefficient boundary conditions modified to account for surface roughness.

**Figure 2:**
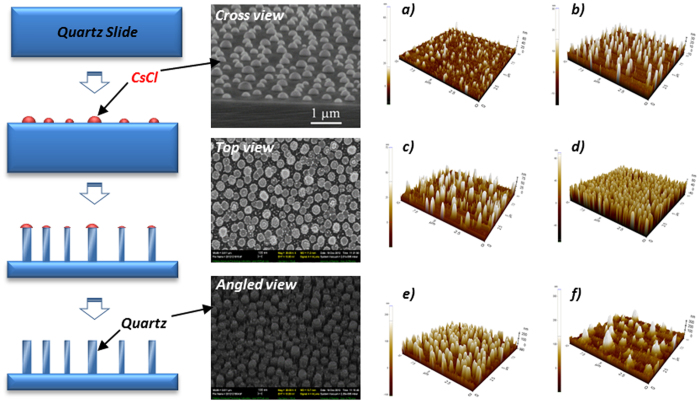
(Left) Schematic of rough surface nano-fabrication; and (Right) AFM profiles of modified quartz-slides after nano-fabrication: representative values of Rmax at (a) 10 nm, (b) 20 nm, (c) 50 nm, (d) 100 nm, (e) 200 nm, and (f) 400 nm.

Schematics of the vacuum-sealed parallel plate chamber system and geometric conceptualization of rough surfaces are shown in Fig. 1(a,b). The deposition surface roughness elements were assumed to be evenly distributed spheres with diameter *a*_*r*_ and a distance of *s* and were vertically assembled as elongated filaments on the plate, which is the bottom surface of the quartz slides. These simplifying assumptions enabled efficient computation^12^ and were possible because of the well-controlled nanofabrication approach that generated uniformly rough surfaces, as confirmed by atomic force microscopy (AFM). The values of *a*_*r*_ and *s* (based on AFM measurements, Fig. 2 (right)) for different roughness features are presented in Table 1. The roughness features on the slide surfaces were comprised of the same materials as smooth slide surfaces; therefore, it was reasonable to assume that they had the same electrical potential, hydrophilic/hydrophobic properties, and Hamaker constant for DLVO interaction energy calculation.

Flow in the parallel plate chamber was assumed to be laminar (due to its low Reynolds number), steady, and incompressible. The suspended colloidal particles (microspheres) passing through the chamber were treated as smooth spherical particles. Particle-particle interactions and blocking effects in the system were negligible, as demonstrated by the quality assurance (QA) experiments; as a result, particle deposition was driven by particle-surface interactions that satisfied the clean bed assumption. Governed by the conservation law, mass transport of one type of particle in suspension at steady state and in the absence of chemical reactions could be expressed as 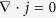 where *j* [(*mol* · *m*)/(*L* · *s*)] is the particle flux vector. The dimensionless particle transport equation and the corresponding boundary conditions were expressed as


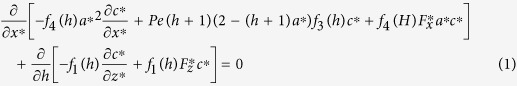


Dimensionless parameters, constants, and the detailed derivation of Equation (1) are presented in the Supplementary Information. Briefly, a*, c* are the dimensionless particle radius and concentration respectively, *Pe* is the Peclet number of the particle, *h* is the distance between the particle and the top of the roughness feature, and 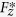
and 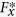 are the scaled force in the *z*- and *x*-directions, respectively. *f*_*i*_*(h)* represents the dimensionless hydrodynamic retardation functions.

As shown in Fig. 1(c), the flow direction in the study system was in the *x*-direction. The upper surface (Boundary 1) was treated as the insulating boundary 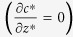, assuming no net particle flux across the upper surface of the parallel plate chamber. The ionic strength in the system and the associated DLVO forces resulted in net attraction between the approaching particles and the bottom surface (Boundary 2), which was treated as a perfect sink (c* = 0). The inlet (Boundary 3) of the simulation domain was regarded as the bulk concentration (c* = 1). The outlet (Boundary 4) of the simulation domain assumed that mass is transported out of the domain by convection only 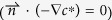. The dimensionless flux of particles (*Sh*) to the bottom surface was calculated at the primary interaction energy minimum, which is usually defined at a cut-off dimensionless distance *δ*, avoiding numerical singularity, so


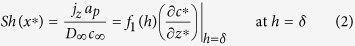


where *j*_*z*_ is the dimensionless deposition flux on the z direction, *a*_*p*_ [m] is the particle radius, *D*_∞_[m^2^/s] is the Stoke-Einstein diffusion coefficient of the particle, and *c*_∞_[mol/L] is the bulk particle concentration.

### Flow field profile

Fully developed flow with a parabolic velocity profile was assumed in the study system; therefore, that the fluid velocity components in the *y*- and *z*-directions were negligible (*v* = 0, *w* = 0). For chemically homogenous and smooth plate surfaces, the no-slip boundary condition is applied to the deposition surface and the undisturbed fluid velocity (*u, v, w*) in the Cartesian coordinate system can be expressed as 
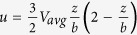
; where *V*_*avg*_ is the average fluid velocity and *b* is half of the chamber height. Notably, when deposition surfaces are rough, accurate description of the flow field profile requires modified boundary conditions. Depending on the absolute height and extent of coverage of the roughness elements, slip or partial-slip boundary conditions may be more approrpiate^42^,^43^. Alternatively, the “effective target surface” can be regarded as a hydrodynamically equivalent smooth plane located between the top and bottom of the roughness elements, as shown in Fig. 1(d), where the no-slip boundary condition is shifted from the top of the roughness elements to the bottom^44^. Based on AFM measurements (Fig. 2), surface roughness coverage was adequately extensive (>80%) in the present study system so that the no-slip boundary condition was accordingly applied at the bottom of the roughness elements. The separation distance between the particles and the deposition surface was modified with dimensionless slip-length *r*_*slip*_ and the flow velocity above the roughness elements (*u*_*sli*p_) was approximated as


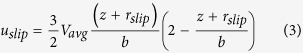


As a result of this adjustment, the velocity component that is parallel to the direction of flow in the chamber was higher for rough surfaces as compared to smooth ones.

### Hydrodynamic retardation functions

In the vicinity of a surface, the velocity of an approaching particle is altered due to the hydrodynamic disturbances caused by the surface; here, the bounding walls of the parallel plate chamber. The dimensionless hydrodynamic retardation functions, *f*_1−4_(*H*), contribute to describing deviations in particle velocity (*u*) from the fluid motion and diffusion tensor (*D*) between the approaching particle and the deposition surface as *u*_*x*_ = *f*_3_(*H*)*u*, *u*_*z*_ = *f*_1_(*H*)*f*_2_(*H*)*v* and *D*_*x*_ = *f*_4_(*H*)*D*_∞_, *D*_*z*_ = *f*_1_(*H*)*D*_∞_ where *H* is the dimensionless surface-to-surface distance^45^,^46^,^47^,^48^. Calculation of the analytical solution for the hydrodynamic retardation functions (HRFs) over the entire simulation domain is computationally intensive for numerical simulation^49^,^50^. In the present study, the hydrodynamic retardation functions (*f*_1−4_) were calculated as a blend of asymptotic solutions described by 
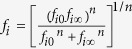
 where *n* is a fitting parameter, *f*_*i*0_ and *f*_*i*∞_ are the analytical solutions for *f*_1−4_ when the dimensionless distance is approaching 0 and infinity. The determined numbers of *n* for *f*_1−4_ were 5, 20, 15, and 10, respectively. Here, the new *f*_1−4_ were reduced to the analytical solution when *H* approaches both asymptotic limits (Table S-1). As compared to a numerical solution^51^ and other solutions previously reported in the literature^33^,^52^,^53^, the new approximated hydrodynamic retardation functions best fit the exact numerical solution with no more than 2% relative difference over the entire simulation domain (Figure S1); they also are simple and computationally efficient relative to other reported approximations.

Deposition surface roughness reduces hydrodynamic retardation^44^,^54^,^55^—models of particle deposition on rough surfaces must reasonably account for this impact. When roughness coverage of surfaces exceeds 50%, the rough surfaces (e.g., such as those in Fig. 1(d)) can be regarded as hydrodynamically equivalent to a smooth surface (no-slip wall) located at the bottom of the roughness feature and the hydrodynamic retardation function that now accounts for surface roughness can be described by


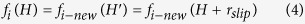


where *H* is the dimensionless distance from the top surface of the roughness *r*_*slip*_ = *r*_*roughness*_/*a*_*p*_ is the dimensionless slip length due to the presence of roughness, and *f*_*i*−*new*_ is the modified hydrodynamic retardation function accounting for the presence of surface roughness. Maximum hydrodynamic retardation occurs at *H* = 0 for a smooth surface and at *H* = *r*_*slip*_ for a rough surface; of course, these functions are also the same as those for a smooth surface if roughness equals 0.

### DLVO interaction energy

Classic DLVO theory has been used to describe the long-range interfacial forces between particles (or particles and surfaces) that are influenced by EDL and VDW forces^24^. The interaction energy between a particle and a rough plate is determined using the modified Derjaguin approach that assumes the DLVO interactions between individual components are additive—this is commonly referred to as the pairwise summation (PS) method. Thus, the total interaction energy between an approaching particle and the bottom of a surface with roughness elements was determined by:


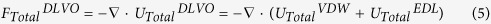


where 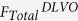 is the sum of the VDW/EDL forces between the particle and surface; 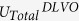, 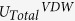 and 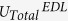 are respectively the total DLVO, VDW, and EDL interaction energies (not forces) accounting for all contributions from the roughness elements and the bottom plane^24^.

### VDW force

According to the PS method, the attractive VDW force, which is the sum of VDW interaction energies between the particle and all of surface roughness elements, is formulated as 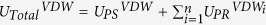, where 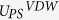 is the VDW interaction energy between the particle and bottom surface and 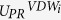 is that between the particle and a roughness element on the surface^14^,^24^. Assuming there is no chemical heterogeneity on the surface and the Hamaker constant between the particle and plate surfaces is not changing, the individual un-retarded VDW interaction energy between two spheres (an approaching particle and a roughness element conceptualized as a stack of spheres; Fig. 1(b)) was expressed as


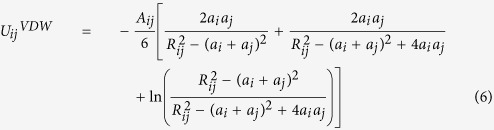


where *R*_*ij*_ is the center-to-center distance between the two spheres and *A*_*ij*_ is the Hamaker constant between the *i*^*th*^ and *j*^*th*^ spherical objects. The approach for calculating sphere–plate (i.e. bottom surface) interaction energy is valid for small separation distances and was described by Gregory (1981)^56^ as 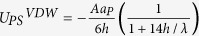 if 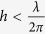.

### EDL force

To evaluate the changes in the interaction energy curves between particles and surfaces after including the effect of deposition surface roughness, measured roughness sizes and surface electrical potentials were used in the numerical simulation. According to Kemps and Bhattacharjee (2005), when *κ*^−1^*a*_*p*_ > 25(*κ*, the inverse Debye length), the total EDL interaction energy between the particle and rough surface can be well approximated by the PS method^57^. Accordingly, the net EDL interaction energy could be approximated as 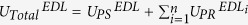; where 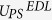 and 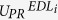 represents the EDL interaction energy between the particle and the plate surface without roughness elements and between the particle and a given roughness element (*i*) on the surface. Based on the classic Hogg-Healy-Fuerstenau expression^58^, the EDL interaction between the approaching particle and spherical component of the roughness asperities could be expressed as


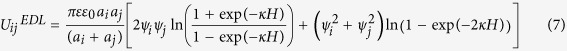


and the EDL interaction energy between the smooth plate and the approaching particle was calculated as


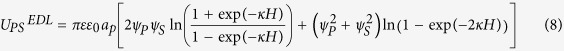


where 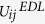 is the EDL interaction energy between two spheres *i* and *j*, *ψ*_*I*_ and *ψ*_*j*_ are the respective electrical potentials, and *H* is the dimensionless surface-to-surface distance.

## Results and Discussion

### Characteristics of quartz slide surfaces with nano-scale roughness

The contact angles of all of the nano-fabricated quartz slides used in the present investigation ranged from 30° to 36°, indicating that the surfaces were hydrophilic (Figure S2). Triplicate AFM measurements of roughness features in a 20 μm × 20 μm section of each slide were employed to obtain the arithmetic average roughness (*R*_*a*_) and the root mean square roughness *R*_*q*_ (Table 1). The AFM images in Fig. 2 (Right) demonstrate that roughness in the shape of hemispherical columns was uniformly distributed over the quartz slide surfaces and that surface coverage on the fabricated slides exceeded 80%. The absolute size of the roughness features (i.e., *R*_*max*_) generally met targeted values at 10, 20, 50, 100, 200 and 400 nm respectively.

### Impacts of roughness on particle deposition—experimental results

**Figure 3:**
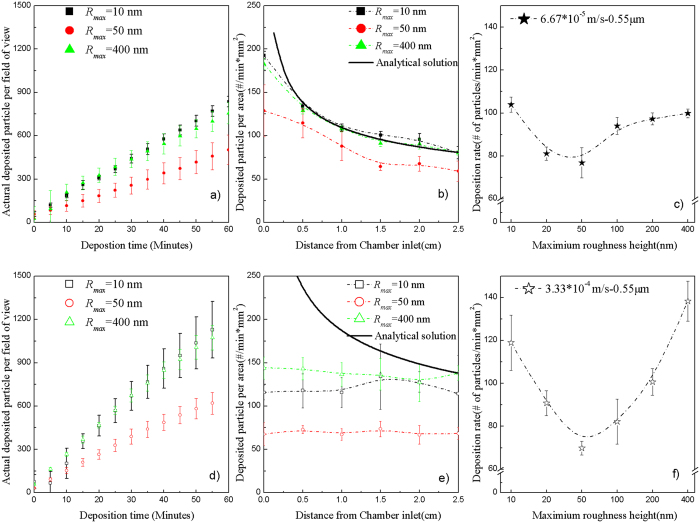
Deposition of 0.55 μm particles at low (6.67 * 10^−5^ m/s) and high (3.33 * 10^−4^ m/s) loading rates. Particle deposition (particles/field-of-view [0.13 mm^2^]) over time at 1.5 cm from the chamber inlet for three levels of surface roughness at (**a**) low and (**d**) high loading rates; particle deposition rate along the flow direction for three levels of surface roughness at low (**b**) and high (**e**) loading rates; and average particle deposition rate for six levels of surface roughness at low (**c**) and high (**f**) loading rates. Error bars represent the standard deviation of five replicate samples.

A non-linear, non-monotonic relationship between deposition surface roughness and particle deposition flux was observed for small particles (<1 μm). This relationship is demonstrated in Fig. 3, which depicts deposition of 0.55 μm particles at low (6.67 * 10^−5^ m/s) and high (3.33 * 10^−4^ m/s) loading rates. Particle deposition at a fixed location (*x* = 1.5 cm from chamber inlet) over time (60 minutes) is presented for three levels of deposition surface roughness (*R*_*max*_ = 10, 50 and 400 nm) at the low and high loading rates in Fig. 3(a,d), respectively. These figures indicate that particle deposition was similar on the smooth and roughest surfaces (*R*_*max*_ = 10 and 400 nm); in contrast, there was significantly less deposition on the moderately rough surface (*R*_*max*_ = 50 nm), regardless of particle loading rate. Notably, during this initial, “clean bed” period, particle deposition was highly linear (*R*^*2*^ > 0.99 for least squares linear regression in all cases). This result is consistent with the confirmatory QA experiments that indicated that there was no particle aggregation in the stock suspension or blocking on the deposition surface (Figures S7 and S8).

Particle deposition within the entire parallel chamber, on surfaces with three levels of roughness, at low and high loading rates is presented in Fig. 3(b,e), respectively. Consistent with Fig. 3(a,d), these figures indicate that particle deposition was similar on the smooth and roughest surfaces (*R*_*max*_ = 10 and 400 nm) and that in contrast, there was significantly less deposition on the moderately rough surface (*R*_*max*_ = 50 nm), regardless of particle loading rate. The difference between the lowest and highest deposition rates was higher in the system with the higher loading rate (Fig. 3(e)) and the deposition curves were relatively flat along the flow direction, as opposed to asymptotically decreasing as in low loading rate system (Fig. 3(b)), thereby underscoring the importance of the flow field profile in the resulting particle deposition profiles. There also was excellent agreement between the analytical solution for particle deposition on smooth surfaces and the observed particle deposition rate within the parallel plate chamber for the relatively smooth surface (i.e., *R*_*max*_ = 10 nm) at the low loading rate (Fig. 3(b)), as would be expected in a system in which diffusion is a dominant particle transport mechanism. Not surprisingly, with increased contribution from convection, the analytical solution for particle deposition on the smooth surfaces did not match the experimental outcomes (Fig. 3(e)). Also as expected, regardless of loading rate, the analytical solution for particle deposition on smooth surfaces did not necessarily match the particle deposition data when there was deposition surface roughness present in the system (Fig. 3(b,e)).

**Figure 4:**
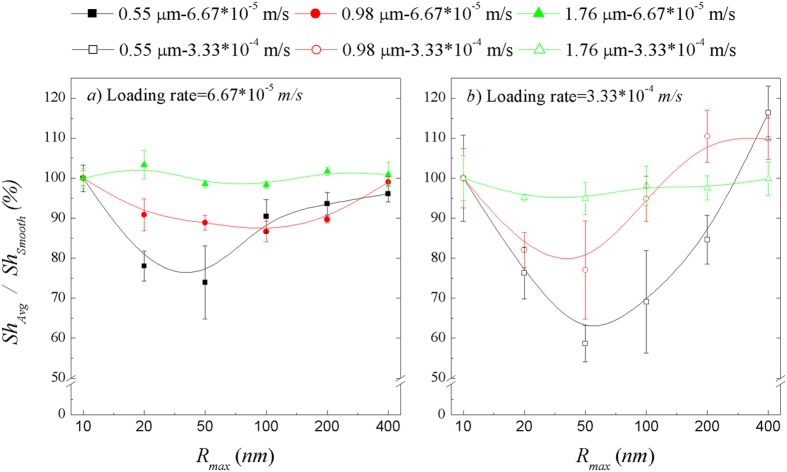
Average particle deposition flux on rough surfaces normalized to that observed on smooth surfaces (*Sh*_*avg*_*/Sh*_*Smooth*_) demonstrating the non-linear, non-monotonic effect of nano-scale surface roughness on particle deposition for various sized particles at various operating conditions. Positive and inverse relationships between particle deposition flux and loading rate and particle size, respectfully are demonstrated. Three particle sizes (0.55, 0.98, and 1.76 μm) at six levels of deposition surface roughness, and at (**a**) low and (**b**) high loading rates are presented. The low and high loading rates were 6.67 * 10^−5^ and 3.33 * 10^−4^ m/s, respectively. Error bars represent the standard deviation of five replicate samples.

Based on the observed deposition rates at individual locations within the chamber, an average deposition rate, *Sh*_exp,*avg*_ was calculated for each of the six deposition surface roughness heights investigated, in which 
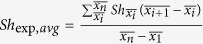
 was the experimentally determined flux at 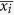 on the deposition curves, n = 5, 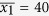 and 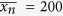. The deposition data at 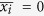 were excluded to minimize potential entrance effects due to non-stabilized flow. These average deposition rates observed at the low and high particle loading rates, are presented in Fig. 3(c,f), respectively. These figures demonstrate a non-linear, non-monotonic relationship between deposition surface roughness and the particle deposition rate. They also demonstrate that there is a critical roughness size (~*R*_*max*_ = 50 nm) or “sag effect” associated with the minimum particle deposition rate. Below the critical roughness size, increases in nano-scale surface roughness result in decreased particle deposition and above it, increases in nano-scale surface roughness result in increased particle deposition. This non-linear, non-monotonic “sag effect” of surface roughness on particle deposition is particularly evident in Fig. 4, where the average normalized deposition flux (i.e., the average particle deposition flux on rough surfaces normalized to that observed on smooth (i.e., *R*_*max*_ = 10 nm) surfaces, *Sh*_exp,*avg*_/*Sh*_*smooth*_) is presented for three particles sizes at two loading rates for six levels of deposition surface roughness.

Clearly, the non-linear, non-monotonic “sag effect” of surface roughness on particle deposition is inversely proportional to particle size (Fig. 4). For example, when particle size increased from 0.55 to 0.98 and then 1.76 μm, the maximum difference in normalized deposition flux across the range of roughness sizes investigated decreased from ~32% to ~13% and ~1%, respectively, at the low loading rate. In contrast, “sag effect” was more pronounced at the higher the loading rate, where the maximum difference in normalized deposition flux was ~42%, ~23% and ~5% for the 0.55, 0.98, and 1.76 μm particles, respectively.

### Impacts of roughness on DLVO interaction energy—numerical results

**Figure 5:**
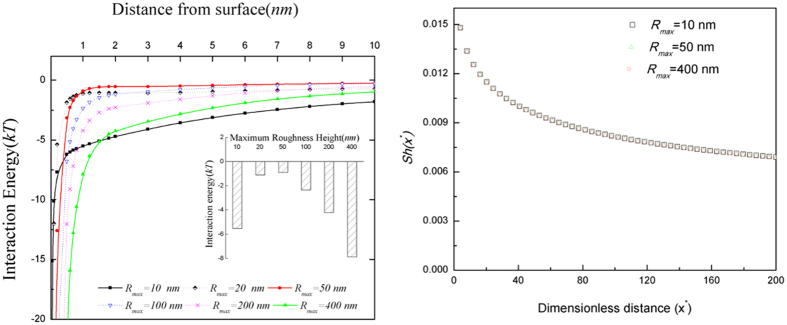
(**a**) DLVO interaction energies calculated between 0.55 μm particles and deposition surfaces with various levels of surface roughness (*R*_*max*_ = 10 nm, 20 nm, 50 nm, 100 nm, 200 nm and 400 nm). The inset figure depicts DLVO interaction energy at a 1 nm distance from the deposition surface and (**b**) the corresponding deposition flux, *Sh* for different sizes of deposition surface roughness.

The DLVO interaction energies between the depositing particles and the deposition surfaces with various roughness sizes were calculated using the PS method proposed above. Nano-scale roughness features on the fabricated slides were described by *a*_*r*_ and *s* using the measurements obtained by AFM (as shown in Fig. 2) and the geometric conceptualization presented in Fig. 1(b). All of the DLVO interaction energy curves followed the same patterns. Representative DLVO interaction energies calculated between the 0.55 μm particles and the deposition surfaces with six levels of surface roughness are presented in Fig. 5(a). As would be expected with a compressed EDL (~0.9 nm), the net DLVO interaction energies for all scenarios were negative, suggesting net attractive forces between the approaching particles and deposition surfaces, regardless of roughness size. Interestingly, deposition surface roughness also had a non-linear, non-monotonic impact on DLVO interaction energy at all separation distances; this is particularly evident in the Fig. 5a inset that shows the DLVO interaction energy at the cut off distance of 1 nm. This relationship alone; however, was inadequate for describing and simulating particle deposition on surfaces with variable roughness (Fig. 5(b)). This is underscored by comparing the relatively wide range of DLVO interaction energies obtained using different roughness sizes (Fig. 5(a)) with the associated deposition profiles that reflect those changes in DLVO interaction energy, but fail to yield any demonstrable differences in particle deposition flux (Fig. 5(b)). This result was not surprising because interaction energy is only a surrogate measure often used to draw inferences about particle deposition flux; however, it is not a direct measure of force acting on the depositing particles. The DLVO force acting on the particles is the derivative of the interaction energy over distance; given that the shapes of the interaction energy profiles in Fig. 5(a) are all similar, the DLVO force for all of the scenarios is also generally similar, thereby leading to the observed similarities in particle deposition (Fig. 5(b)).

### Agreement between numerical solution and experimental data

**Figure 6:**
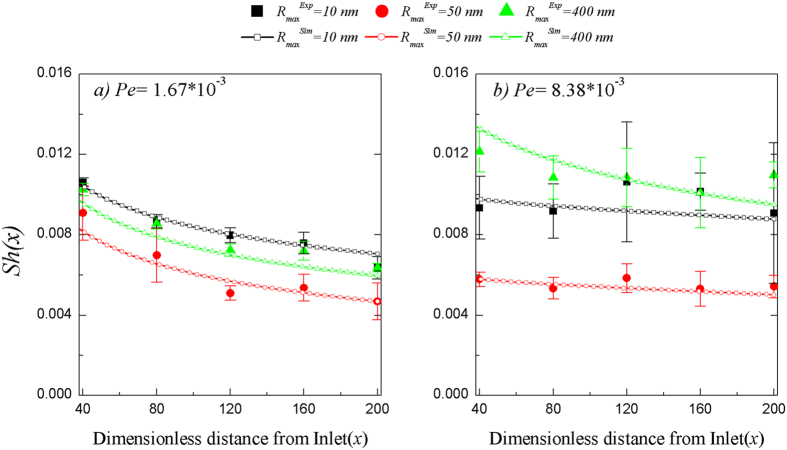
Demonstration of good model agreement by comparison of dimensionless particle deposition flux determined experimentally (*Sh*_*exp*_) and by numerical simulation (*Sh*_*sim*_) for 0.55 *μ*m particles at three levels of surface roughness at loading rates of (a) 6.67 * 10^−5^ and (b) 3.33 * 10^−4^ m/s, respectively. The data in (**a**,**b**) are the data depicted in Fig. 3(b,e), respectively. Error bars represent the standard deviation of five replicate samples.

The Convective-Diffusion model developed herein provides a comprehensive framework for describing the hydrodynamic impacts of nano-scale surface roughness on particle deposition by unifying hydrodynamic forces (using the most current approaches for describing flow field profiles and hydrodynamic retardation effects) with appropriately modified expressions for DLVO interaction energies, and the gravity force. The simulation results using the developed Convective-Diffusion model were compared to the experimentally obtained particle deposition data (Fig. 6). In this figure, the discrete data points and associated standard deviations represent experimental observations (*Sh*_*exp*_) every 0.5 cm along the surface (corresponding to dimensionless x* of 40) and the lines represent the numerical simulation results. Comparison of dimensionless particle deposition flux determined experimentally (*Sh*_*exp*_) and by numerical simulation (*Sh*_*sim*_) for 0.55 μm particles at three levels of surface roughness with loading rates of (a) 6.67 * 10^−5^ and (b) 3.33 * 10^−4^ m/s, respectively indicated good model agreement. The experimental data in Fig. 6(a,b) are the data depicted in Fig. 3(b,e), respectively. Particle deposition data at the entrance to the chamber (*x* = 0 cm) were excluded from the comparison because of potential entrance effects. Good model agreement was also observed with the experimentally obtained 0.98 μm and 1.76 μm particle deposition data (Figures S3 and S-4, respectively).

### Mechanistic insights

It was demonstrated herein that the combination of hydrodynamics (i.e. flow field profiles and hydrodynamic retardation) and gravity in combination with appropriate representation of the DLVO force could reasonably explain the observed impact of nano-scale surface roughness on particle deposition (shown in Fig. 6). Although chemical heterogeneity, the secondary energy minimum, and hydrophilic properties are commonly recognized, chemically-based mechanisms that can influence particle deposition behavior, they did not result in the non-linear, non-monotonic relationship in particle deposition that was observed in the present investigation. The experimental design and operational conditions utilized in the present investigation minimized and excluded confounding effects of surface chemical heterogeneities, surface hydrophilic properties, and the secondary energy minimum between approaching particles and target surfaces. The use of 100 mM KCl compressed the electrical double layer around the particles and contact surfaces, thereby minimizing the impact of the secondary energy minimum. Contact angle measurements on the contact surfaces did not indicate substantial differences in surface hydrophilic properties. Microscopic evaluation of all modified quartz slide contact surfaces did not reveal any indications of surface chemical heterogeneities; moreover, extensive particle aggregation and/or blocking were not observed.

As demonstrated above (Fig. 5), the widely accepted variation of interaction forces (i.e. the DLVO force) between approaching particles and a target surface also provided limited explanation of the observed non-linear, non-monotonic relationship between surface roughness and particle deposition. The relatively insignificant impact of changes in DLVO interaction energy on particle deposition was not surprising because interaction energy is not a direct measure of force acting on depositing particles; it is only a surrogate measure often used to draw inferences about particle deposition flux. The interaction energy profiles in Fig. 5 were generally similar to one another in shape and therefore yielded similar DLVO force (i.e. derivative of the interaction energy between the particles and the surface over distance). Thus, the particle deposition flux profiles obtained when only DLVO interactions were considered were also similar to one another, despite changes in nano-scale surface roughness size and in contrast to the experimental data.

**Figure 7:**
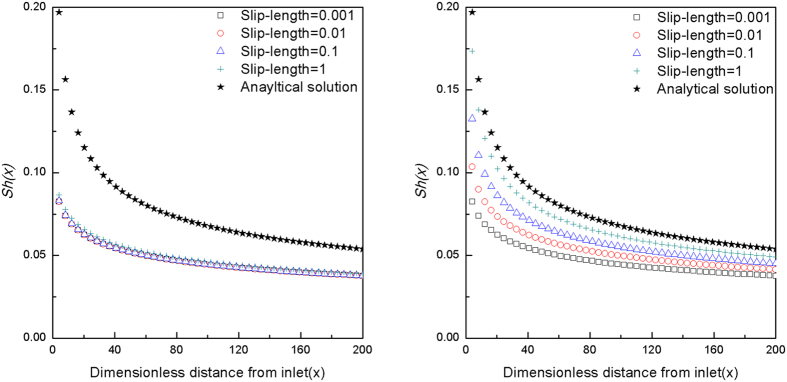
Particle deposition flux calculated using the analytical solution and different slip-lengths (i.e. dimensionless roughness sizes) for (a) flow field modification and (b) hydrodynamic retardation functions modification for 1.0 μm particle size, *Pe* = 0.1, and *Gr* = 0.

The present investigation demonstrated that hydrodynamic mechanisms (i.e. flow field characteristics and hydrodynamic retardation) combined with gravity and appropriate DLVO force characterization could reasonably describe the experimental results (Fig. 6). This mechanistic conclusion can be clearly observed by a systematic sensitivity analysis in which a hydrodynamically equivalent plane on the bottom of the roughness elements was used as the “effective target surface” to numerically represent the impact of surface roughness on particle deposition (Fig. 7). Specifically, the effect of surface roughness (represented in dimensionless form by slip length) on the flow field and hydrodynamic retardation functions was evaluated individually with exclusion of DLVO interaction and gravity by setting the Hamaker constant, electrical potential, and gravity effect on the particles to zero (Fig. 7a,b, respectively). Notably, comparison of Fig. 7a,b underscores that while deposition flux was relatively insensitive to flow field changes (Fig. 7a), hydrodynamic retardation contributions were critical to the good agreement between the experimental data and model outcomes presented in Fig. 6; particularly at distances within one particle radius from the contact surface (Fig. 7b).

It should be noted that the effective slip-length used for approximating the flow field and hydrodynamic retardation functions might not be the same value, depending on the physical and chemical properties of approaching particles and contact surfaces. As well, other factors such as short range forces, shear lift, and particle rolling may also contribute to further explaining the observed non-linear, non-monotonic relationship between deposition surface roughness and particle deposition. Further investigations are needed to explore these relationships.

## Conclusions

A non-linear, non-monotonic relationship between deposition surface roughness and particle deposition flux, particularly for small particles (<1 μm), in absence of an energy barrier was rigorously demonstrated and a critical roughness size associated with minimum deposition flux or “sag effect” was identified. When roughness size was less than the critical value, particle deposition decreased with increased roughness. Particle deposition increased with increased roughness size when it was above the critical value. The non-linear, non-monotonic relationship was modeled well numerically using the developed Convective-Diffusion model and experimentally validated. Of course, it is recognized that consideration of other system factors (e.g., short range forces, surface heterogeneity, hydrophobicity, etc.) will also influence particle deposition behavior. Nonetheless, with applicability in areas ranging from drinking water treatment^30^ to health screening^6^,^7^ and semiconductor manufacturing^10^, a better ability to quantitatively describe the deposition of colloid- and nano-scale particles on rough surfaces is an essential step toward describing and predicting these phenomena in real natural and engineered environments. Our results incontrovertibly show that there can be potentially significant non-linear, non-monotonic effects of nano-scale deposition surface roughness on particle deposition in the absence of an energy barrier, a condition representative of many engineered and natural systems. These effects depend on several system characteristics including the particle loading rate and size, as well as deposition surface roughness size. Moreover, these effects are particularly significant for smaller (<1 μm diameter) particles such as viruses, bacteria, cells, and industrial nano-particles. The comprehensive framework developed herein provides an important first step toward quantitatively describing deposition of colloid- and nano-scale particles on rough surfaces and a foundation for further describing the impacts of more complicated scales of deposition surface roughness on particle deposition.

## Materials and Methods

### Colloidal particles

Carboxylated fluorescent (441 nm excitation, 486 nm emission) polystyrene microspheres (Polysciences Inc., PA) with a diameter of 0.55 ± 0.017 μm, 0.98 ± 0.047 μm and 1.76 ± 0.31 μm were utilized; their concentrations in the stock suspensions were 3.64 × 10^11^, 4.55 × 10^10^ and 5.68 × 10^9^ particles/mL, respectively. The reported density of the microspheres was 1.045 ± 0.005 g/mL. To minimize the EDL force, the microspheres were suspended in 100 mM KCl, which resulted in an ~0.9 nm thick EDL layer. The microsphere suspensions were sonicated for 30 minutes before each experiment to ensure particle disaggregation. Electrokinetic properties of the microspheres were obtained using dynamic light scattering (DLS) (Zetasizer nano range, Malvern, UK); measured electrophoretic mobility was converted to zeta potential using Smoluchowski’s equation. Colloidal particle size distribution measurements obtained by DLS before and after all deposition experiments confirmed negligible microsphere aggregation in the stock suspensions during the experiments.

### Quartz slide pretreatment

Quartz microscope slides of initial surface roughness were utilized (Ted Pella Inc., Redding, USA). The slides were sonicated in acetone, cleaned using the RCA 1 method^59^, and then rinsed with isopropyl alcohol. They were then rinsed with DI water and blown dry with pure N_2_ gas. Briefly, After the nano-fabrication process, the modified slides were rinsed with Milli-Q^TM^ water and then analytical grade acetone, and were dried with N_2_ gas.

A nano-fabrication method using CsCl self-assembly^60^ was employed to generate different roughness sizes on the slide surfaces as demonstrated in Fig. 2 (Left). CsCl was evaporated at a pressure of 6 *μ*Torr in a humid chamber and deposited onto the slide surfaces, forming isolated hemispheres by kinetic dissolution and deposition at the solid/solution boundary^60^. The slides were then placed in a chamber at 21 °C with a relative humidity of 22% for 10 minutes. The spherical shapes of thin layers of CsCl (10 nm) were used as a mask to pattern the quartz surface using reactive ion etching (RIE) with a constant etch rate of 25 nm/minute, aiming to create individual maximum surface roughness heights (*R*_*max*_) at targeted sizes.

Streaming potential analysis (Surpass Anton Paar, VA, USA), contact angle measurement (Axisymmertic drop shape analysis-profile, University of Waterloo, Canada), and atomic force microscopy (AFM) (XE-NSOM, Park Systems, Korea) were used to characterize modified quartz slide zeta potential, hydrophilic properties, and surface topology (Table 1). Because each slide surface was etched rather than coated, the composition and chemical properties of the modified and unmodified slides remained consistent, with negative zeta potential (Table 2).

### Experimental Setup

Colloidal particle deposition experiments were conducted in a vacuum-sealed parallel plate flow chamber (GlycoTech, MA, USA) with inner dimensions of 4 × 1 cm and a height of 250 *μ*m (Fig. 1(a)). The parallel plate chamber was firmly bonded on the quartz slides and installed on the stage of an automated inverted fluorescence microscope (Eclipse Ti, Nikon, Canada). During each experiment, images were continuously acquired at nine locations along the flow direction from the inlet to the outlet of the chamber, with five replicated measurements in the cross-flow direction at each location (Figure S5). All cross-flow measurements at each location were treated as replicates because the distances between points were several orders of magnitude greater than the deposited particle size, so each point could be considered independent and obtained at identical experimental conditions.

During each experiment, the parallel plate chamber was first primed to prevent bubble entrapment. Specifically, it was placed in a vertical orientation and particle free 100 mM KCl was pumped through it using a syringe pump (ID: 55–333, Harvard Apparatus, Canada) at a flow rate of 100 μL/min for 15 minutes. The chamber was then placed on the monitoring stage (PRIOR Scientific, MA, USA) and the flow rate was adjusted to the desired value for 30 minutes to ensure steady flow conditions within the chamber. After flow was stabilized, the colloidal particle suspension was pumped through the chamber at rates of 10 or 50 *μ*L/min; corresponding to mean loading rates of 6.67 * 10^−5^ and 3.33 * 10^−5^ m/s, respectively. All experiments were conducted at temperatures between 22 and 24 °C. Image analysis was used to evaluate particle deposition on the slide surfaces. An image analysis program was developed in MatLab^®^ to enumerate deposited particles and confirm that they remained at fixed locations after being deposited. Microsphere deposition on the quartz slides was imaged at 400× every 5 minutes during the 60-minute duration of each experiment. A relatively long exposure time (1 second) was used to distinguish between deposited and moving particles. Moving particles appeared as streaks, whereas deposited particles appeared as discrete spheres that remained at a fixed location in subsequent images.

### Image analysis

Given an image *I*, local Hessian matrices Φ were computed for each pixel, 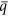 as follows: 
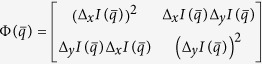
 where ∆_x_ and ∆_y_ denote discrete approximations of derivatives with respect to the x and y directions, respectively. The local Hessian determinants γ were computed based on the local Hessian matrices Φ as 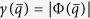, where |·| denotes the matrix determinant. A set of possible cell centroids (denoted by Ω) were identified using non-maximal suppression, and an empirical probability density function of the matrix determinants of the possible cell centroids (denoted by P(*γ*)) was then constructed, and the set of pixels corresponding to actual cell centroids (Ω_*cell*_) was identified using statistical analysis of the constructed probability density function P(γ) as the set of all possible cell centroids in Ω with Hessian determinants that were greater than *γ*_*t*_: 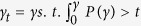 where *t* = 0.5 in this study. Aggregated and moving particles were excluded by setting the threshold for particle size and shape in the enumeration code. To confirm the accuracy of the microsphere enumeration program, selected images were manually counted and compared to the counts obtained with the automated system.

The deposition rate (number of colloidal particles per unit area per unit time or deposition flux *J*) is described by the slope of particle accumulation over time. The dimensionless Sherwood number (*Sh*) determined from experimental results can be calculated as by 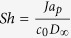 where 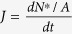 [(mol · m)/(L · s)]^19^,^31^. Here, *N* is the number of colloids deposited at each location during a given time interval, *t* [*s*]; *A* [*m*^*2*^] the microscopic image area; and *C*_*0*_ [*mol/L*] is the initial colloidal particle concentration. When particles deposit during the initial or “clean bed” period, particle-particle interactions in the colloidal suspension, multilayer-colloidal deposition, and blocking effects are negligible. Thus the dominant factor that governs particle deposition is particle-surface interaction^31^, which theoretically leads to a linear relationship of deposited particles per area over time. In the absence of external forces including gravity, interception, and colloidal and hydrodynamic interactions, the analytical solution for Sherwood number (*Sh*_*0*_) representing the ratio of convective to diffusive mass transport is 
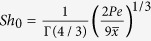
 Here, 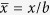, where *x*[*m*] is the distance to the inlet from the point of entry in the parallel plate chamber.

### Quality Assurance (QA) experiments

To ensure observed results were exclusively attributable to deposition surface roughness, potential confounding factors including variation in influent concentration, particle aggregation/blocking due to high ionic strength, and washing protocol for slide cleaning were excluded or minimized by using the developed experimental procedures and/or by the large number of experimental data collected (1125 images per experiment). The results of the QA experiments are briefly discussed and presented in the Supporting Information (Figures S6, S7, and S8).

### Numeral Implementation

The numerical solution of the Convective-Diffusion Equation with four boundary conditions was obtained using the finite element method (FEM) in the commercial simulator COMSOL^®^ 3.5a-Convective and Diffusion Module (COMSOL, Inc., Canada). Exponentially distributed quadrilateral meshes were utilized to discretize the computational domain. Highly refined meshes were used for the regions with a high concentration gradient or a large tensor of applied forces. The size of the smallest mesh in Domain 1 was 10^−5^ in dimensionless height, a value three orders of magnitude smaller than the Debye length at the ionic strength used in the study system. This enabled accurate determination of any flux change due to the high concentration gradient or large force tensor in the vicinity of the bottom surface. To validate the numerical solution obtained by simulation, the solutions developed herein are compared to the analytical solutions. The numerical solutions obtained using the developed model were in excellent agreement with the analytical solutions for all conditions investigated (Figure S9).

## Additional Information

**How to cite this article**: Jin, C. *et al.* Non-linear, non-monotonic effect of nano-scale roughness on particle deposition in absence of an energy barrier: Experiments and modeling. *Sci. Rep.* **5**, 17747; doi: 10.1038/srep17747 (2015).

## Supplementary Material

Supplementary Information

## Figures and Tables

**Table 1:** Quartz slide characteristics.

R_max_ *(nm)*	Measured R_a_ *(nm)*	Measured R_q_ *(nm)*	Water Contact angle *(deg)*	Zeta-potential *(mV)*	a_r_ *(nm)*	s *(nm)*
10	4.3 ± 0.5	5.6 ± 0.5	32.9 ± 1.3	−18.3 ± 3.5	1.2	50
20	8.9 ± 0.2	9.8 ± 0.3	32.2 ± 2.3	−21.5 ± 6.7	3.5	800
50	10.7 ± 1.8	14.6 ± 1.8	36.9 ± 0.3	−18.2 ± 5.1	9	800
100	30.3 ± 2.9	35.9 ± 3.3	35.1 ± 2.3	−14.9 ± 5.8	16	400
200	53.5 ± 7.6	65.1 ± 6.4	30.8 ± 2.3	−16.6 ± 7.4	30	800
400	60.7 ± 9.1	81.4 ± 7.8	30.7 ± 4.6	−15.3 ± 5.2	50	2000

**Table 2:** Physical/chemical characterization of colloidal particles in 100 mM KCl.

Particle Diameter (*μ*m)	Measured Particle size (*μ*m)	Zeta-potential (*mV*)	Initial concentration (*particle/ml*)	Gravity number [−]
0.525	0.55 ± 0.017	−18.5 ± 6.3	7.28 * 10^7^	0.00257
1.03	0.98 ± 0.047	−24.9 ± 3.1	5.71 * 10^7^	0.0264
1.76	1.76 ± 0.31	−21.6 ± 2.2	1.14 * 10^7^	0.273
