# Supplementary material for: Non-linear, non-monotonic effect of nano-scale roughness on particle deposition in absence of an energy barrier: Experiments and modeling
Source: Sci Rep. 2015 Dec 11;5:17747. doi: 10.1038/srep17747 (PMC4675987; doi:10.1038/srep17747)
Supplement: Supplementary Information [file srep17747-s1.pdf]

Supporting information for

Non-linear, non-monotonic effect of nano-scale roughness on particle deposition in absence of an energy barrier: Experiments and modeling

Chao Jin [a], Tomasz Glawdel [b,c], Carolyn L. Ren [c] and Monica B. Emelko [a]*

[a] Department of Civil and Environmental Engineering, University of Waterloo,

200 University Ave W., Waterloo, ON, N2L 3G1, Canada.

[b] Xagenic Inc., 55 York Street, Suite 1000, Toronto, Ontario, M5J 1R7, Canada.

[c] Department of Mechanical and Mechatronics Engineering, University of Waterloo,

200 University Ave W., Waterloo, ON, N2L 3G1, Canada.

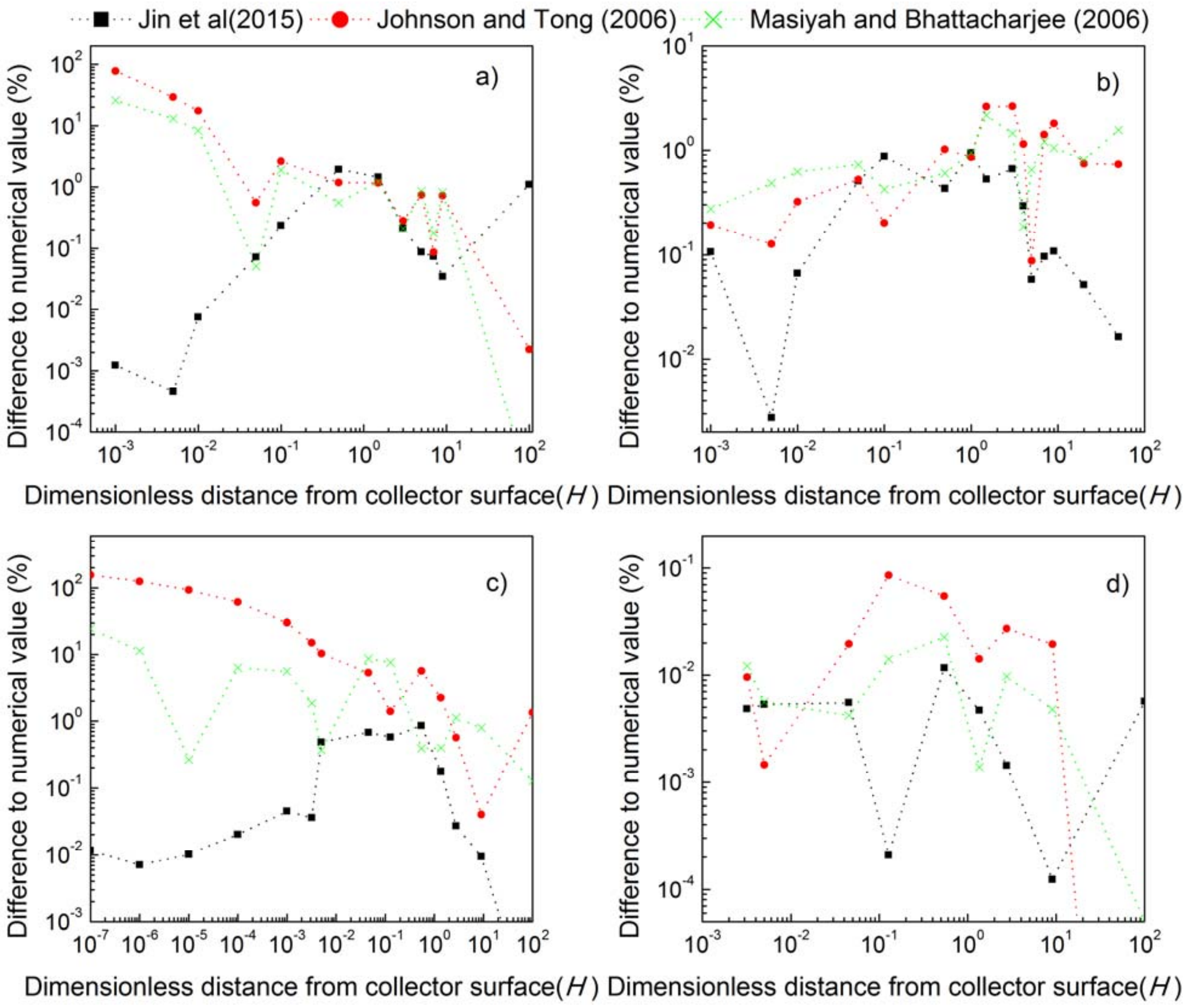

Figure S-1. Fit of the developed and reported[1-3], hydrodynamic retardation functions $f_1(H)$, $f_2(H)$, $f_3(H)$ and $f_4(H)$ (a-d) to the numerical solution reported by Elimelech (1990).

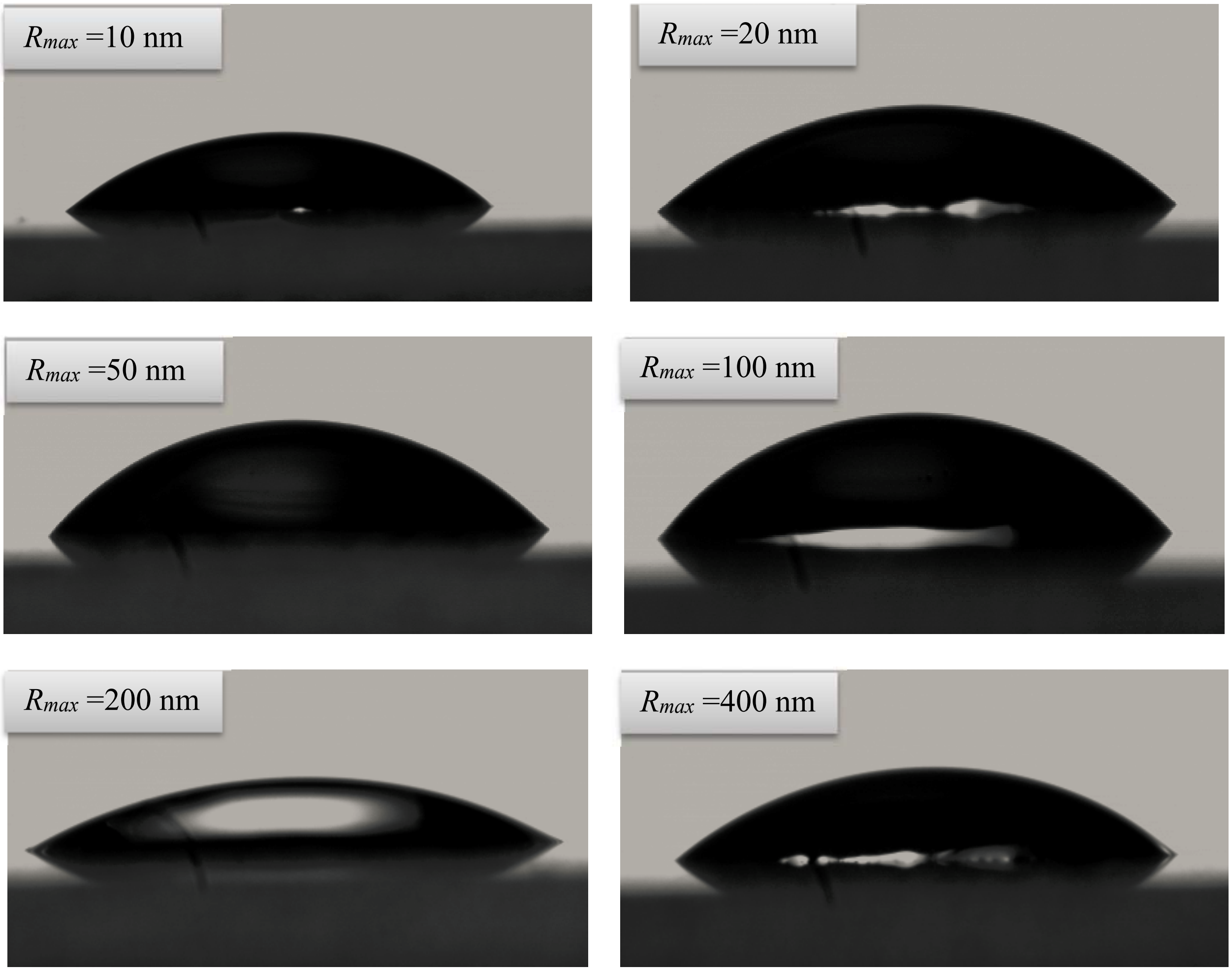

Figure S-2. Examples of images used for contact angle measurement with 100 mM KCl for different surface roughness sizes (R_{max} = 10, 20, 50, 100, 200 and 400 nm).

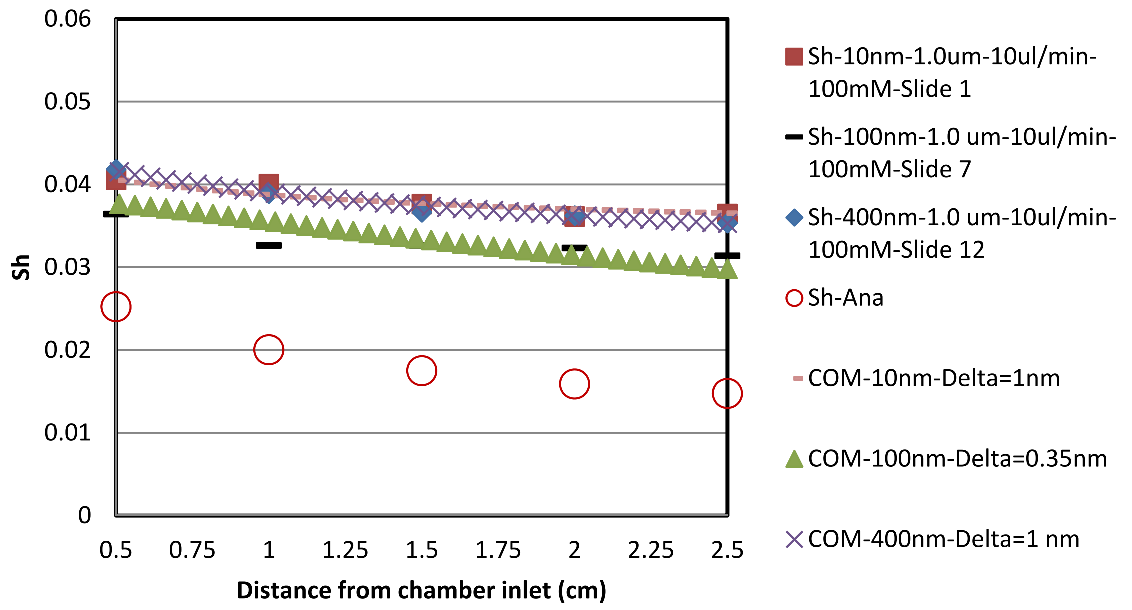

Figure S-3. Comparison of dimensionless particle deposition flux determined experimentally and by numerical simulation for 0.98μm particles at three levels of surface roughness and at loading rates of 6.67*10-5 m/s.

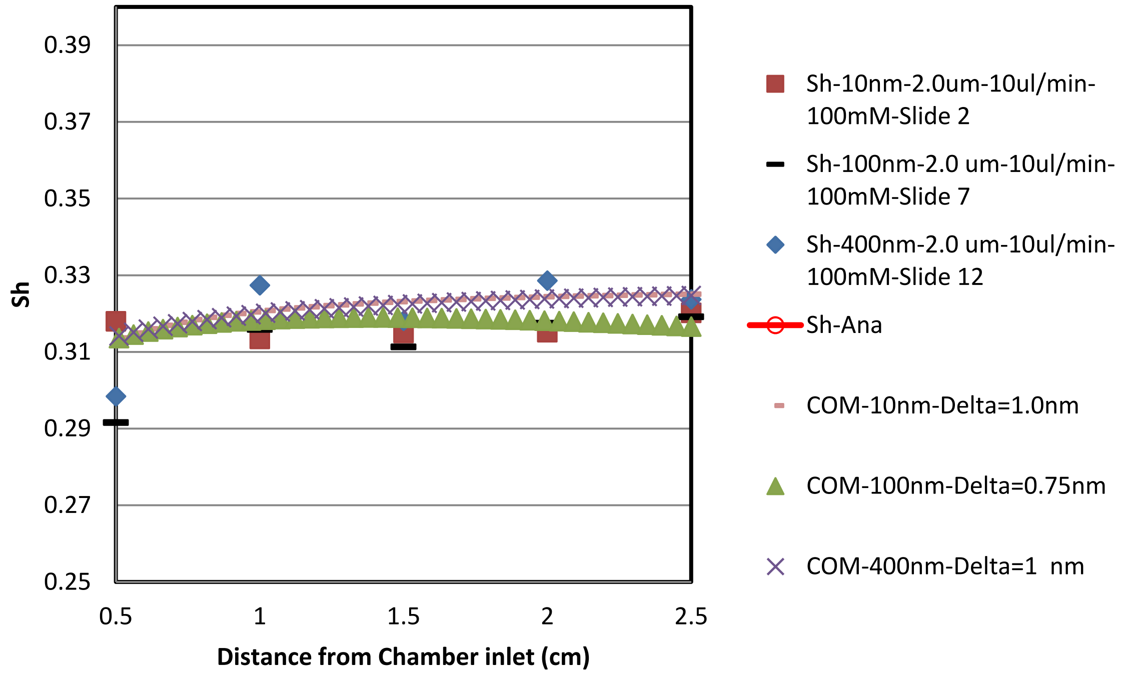

Figure S-4. Comparison of dimensionless particle deposition flux determined experimentally and by numerical simulation for 1.78 μm particles at three levels of surface roughness and at loading rates of 6.67*10-5 m/s.

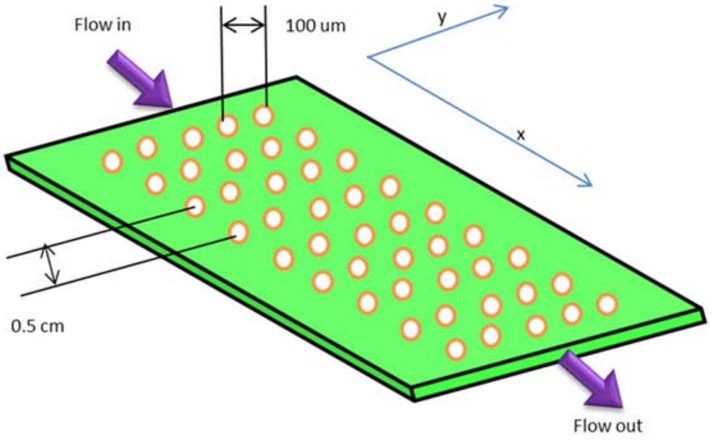

Figure S-5. Sampling protocol on a nano-fabricated quartz slide in the parallel plate chamber.

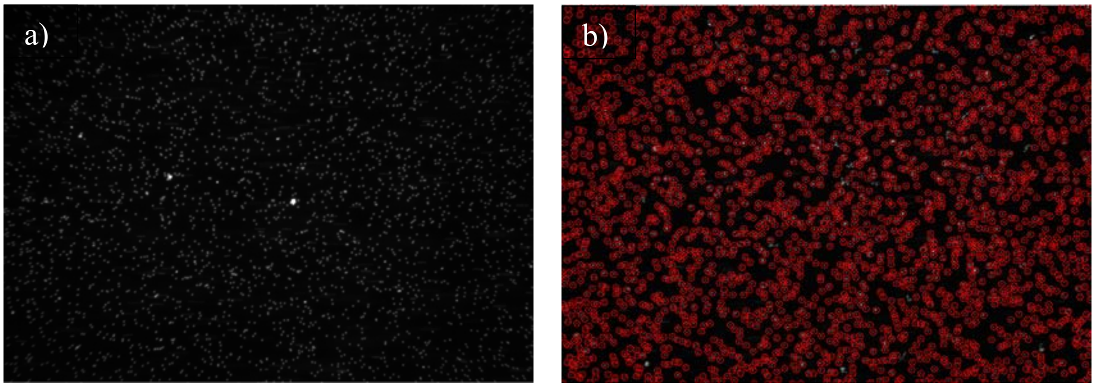

Figure S-6. Representative images of (a) an initial image of deposited particles and (b) an enumerated image depicting microsphere deposition on a nano-fabricated quartz slide.

To confirm the accuracy of the microsphere enumeration program, selected images were manually counted and compared to the counts obtained with the automated system. To ensure the maximum accuracy, the program was designed to a) confirm that particles remained at a fixed location after being deposited; b) count particles accurately despite lens curvature, which meant counting in blurred image areas or areas containing over-bright particles due to lens curvature; and c) exclude aggregated particles from the count. Local Hessian matrices were computed for

each pixel in each image, and the determinant of each matrix was calculated. A probability density function of the matrix determinants was then constructed and the cell centroids were identified using non-maximal suppression and statistical analysis on the constructed probability density function. Aggregated and moving particles were excluded by setting the threshold for particle size and shape in the enumeration code. To confirm the accuracy of the microsphere enumeration program, selected images were manually counted and compared to the counts obtained with the automated system. Representative initial and enumerated images are presented in Figure S-6(a) and (b), respectively.

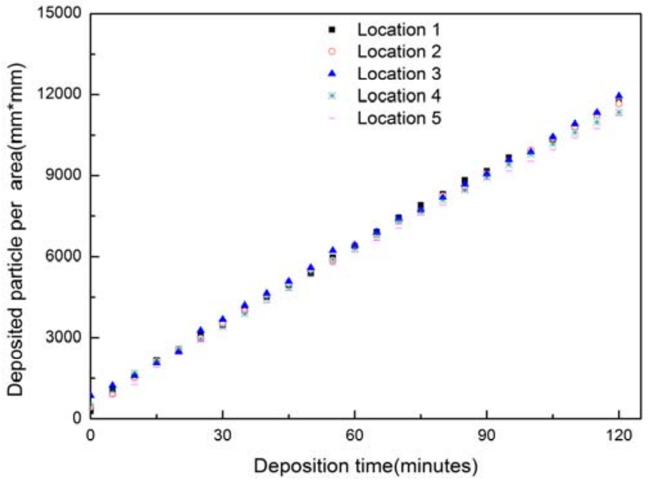

Figure S-7. Representative plot of the number of polystyrene particles (here 0.55 μm) deposited over time at five locations across the width of the parallel plate chamber.

To determine particle flux to the target surface, the number of deposited particles at each location was tracked over time. A representative plot of the cumulative number of attached particles per unit area (image) as a function of time is presented in Figure S-7. This figure demonstrates that the number of deposited particles increased linearly with time within the first two hours, indicating non-impeded deposition (i.e., negligible blocking). Excluding the first 10 minutes

during which the system was primed, a linear relationship (R^2 >0.999) in particle deposition over time was observed at all locations during the experiments. The slope of each individual curve was used to assess particle deposition (*J* and *Sh*).

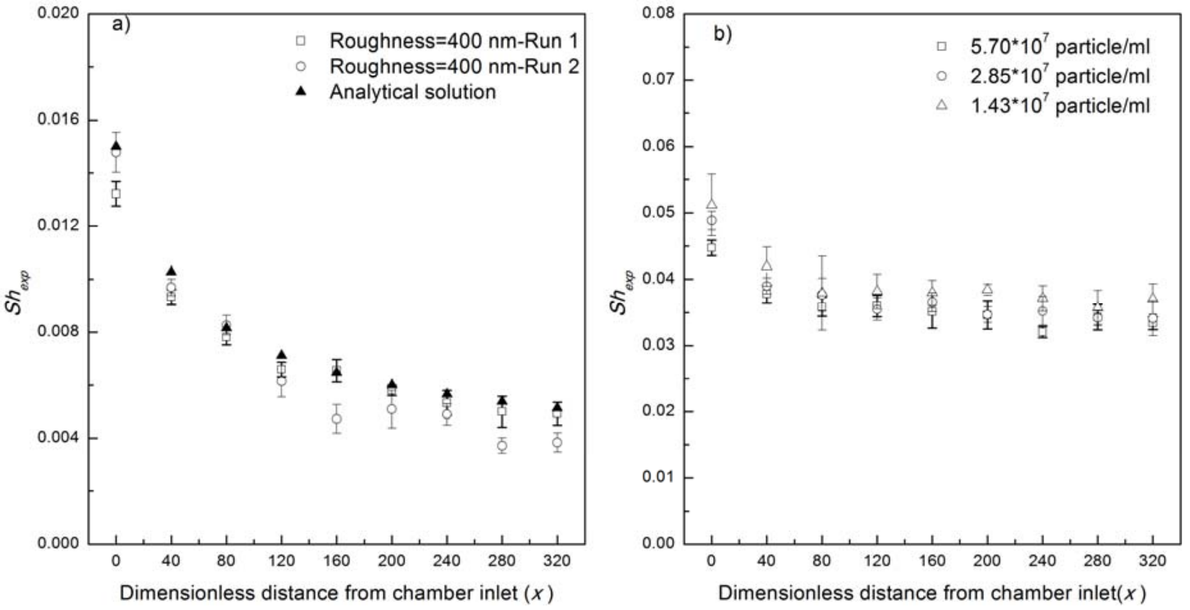

Figure S-8. Representative deposition curves for (a) cleaning test with operational conditions of 400 nm roughness, colloidal particle diameter: 0.55 μm, initial particle concentration: 7.28*10^7 particle per ml; and b) concentration test using smooth slides, colloidal particle diameter: 0.98 μm, initial particle concentrations: 5.7*10^7, 2.85*10^7 and 1.425*10^7 particles/mL, flow rate: 10μL/min, ionic strength: 100mM KCl, pH~6.9, and temperature: 22˚C. The error bars represent the standard deviation from the five replicated results.

Convective interaction is also ignored based on an evaluation of the relative contributions of Brownian convection and diffusion, which is represented by the dimensionless number, *N*:

$$N = \frac{3Vb}{2D_\infty} = \frac{Peb^3}{a^3} \qquad (1)$$

Here, *V* [m/s] is the average flow velocity, *b* [m] the half channel height, a_p [m] the particle radius, D_∞ [m^2/s] the particle diffusion coefficient in bulk solution, and *Pe* the dimensionless

number that evaluates the relative contributions of convection and diffusion and is calculated from the Stokes-Einstein Equation:

$$Pe = \frac{3Va_p^3}{2b^2 D_\infty} \tag{2}$$

Five measurements were collected at each of X locations along the width of the slide (as depicted in the Figure S-8(a)—each of these deposition experiments was conducted in duplicate. No differences in particle deposition (*Sh*) from the experiments conducted before and after slide cleaning were observed. These results demonstrated that the cleaning method used during these and subsequent experiments effectively removed retained particles and returned the quartz slide surfaces to a "clean bed" condition so that they could be re-used. The duplicate deposition data were compared to results obtained using the analytical solution for particle deposition (Equation (3)). As the selected particle size was relatively small (0.55 μm), *N* was 17402 (>>1.0), indicating that this key assumption of the analytical solution was satisfied for the experimental conditions applied herein. As demonstrated in Figure S-8(a), excellent agreement between the experimental data and the analytical solution for dimensionless particle deposition (*Sh*) was obtained, as would be expected when the assumptions of the analytical solution were met.

Particle deposition (*Sh*) at the nine sampling locations along the distance from the chamber inlet to the outlet is presented in Figure S-8(b). Particle deposition (*Sh*) after concentration normalization did not vary between the duplicate experiments. Moreover, the data demonstrated that the initial particle concentration did not impact particle flux to the surface; accordingly a higher initial particle concentration could be utilized to shorten the duration of experiments.

The QA/QC experiments demonstrated that neither the quartz slide washing protocol, particle aggregation due to high ionic strength in the stock suspension, nor influent particle concentration

affected clean bed conditions on the quartz slides between experiments. The large number of experimental data (1125 images per experiment) exhaustively demonstrated the accuracy of the data. Accordingly, these experiments contributed to ensuring that the results obtained during the actual experiments incontrovertibly demonstrated surface rough impacts on particle deposition.

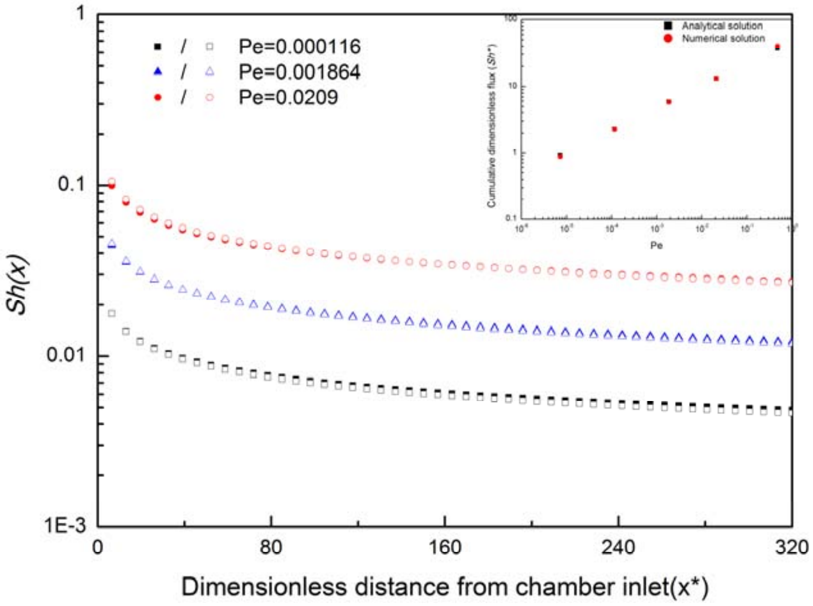

Figure S-9. Developed numerical solution model validation with the analytical solution.

In the absence of external forces including gravity, interception, and colloidal and hydrodynamic interactions, the analytical solution for Sherwood number (*Sh*) representing the ratio of convective to diffusive mass transport is

$$Sh = \frac{1}{\Gamma(4/3)}\left(\frac{2Pe}{9\bar{x}}\right)^{1/3} \tag{3}$$

Here, *Sh* is the dimensionless flux of particles to the surface and $\bar{x} = x/b$, where x[m] is the distance to the inlet from the point of entry in the parallel plate chamber.

Table S-1. Analytical solutions for the hydrodynamic retardation functions when the dimensionless distance (H) approaches 0 or ∞.

Hydrodynamic retardation functions	*When H approaches 0*	*When H approaches* ∞
$f_1(H)$	$H\left(1+\frac{1}{5}H\ln\left(\frac{1}{H}\right)+0.971264H\right)^{-1}$	$\left(1+\frac{9}{8(H+1)}\right)^{-1}$
$f_2(H)$	3.2295	$\left(1-\frac{9}{8(H+1)}\right)^{-1}$
$f_3(H)$	$\left(0.66-0.2693\ln(H)\right)^{-1}$	$1-\frac{5}{16(H+1)^3}$
$f_4(H)$	$-\left(0.5\ln(H)-1\right)^{-1}$	$1-\frac{9}{16(H+1)}$

Table S-2. Dimensionless parameters and constants used in the scaled convection-diffusion equation.

Dimensionless parameter	*Expression*
Scaled surface to surface distance	$H=z^*-1$
Scaled particle radius	$a_p^*=\frac{a_p}{b}$
Scaled distance along the flow direction	$x^*=\frac{x}{b}$
Scaled vertical distance	$z^*=\frac{z}{a_p}$
Scaled concentration	$c^*=\frac{c}{c_\infty}$
Dimensionless Peclet number	$Pe=\frac{3}{2}\frac{V_{avg}a_p^3}{b^2D_\infty}$
Diffusion coefficient	$D_\infty=\frac{k_bT}{6\pi\mu a_p}$
Scaled gravitational force	$Gr=\frac{\vec{F}_g * a_p}{k_bT}$
Scaled DLVO force	$DLVO=-\frac{d\Psi}{dy}*\frac{a_p}{k_bT}$

References

1. Masliyah, J. H. & Bhattacharjee, S. in *Electrokinetic and colloid transport phenomena* (John Wiley & Sons, 2006).

2. Johnson, W. P. & Tong, M. Observed and simulated fluid drag effects on colloid deposition in the presence of an energy barrier in an impinging jet system. *Environ. Sci. Technol.* **40**, 5015-5021 (2006).

3. Elimelech, M. Particle deposition on ideal collectors from dilute flowing suspensions: Mathematical formulation, numerical solution, and simulations. *Separations Technology* **4**, 186-212 (1994).
